# Cloning a novel endo-1,4-β-d-glucanase gene from *Trichoderma virens* and heterologous expression in *E. coli*

**DOI:** 10.1186/s13568-016-0282-0

**Published:** 2016-11-09

**Authors:** Rong Zeng, Qiao Hu, Xiao-Yan Yin, Hao Huang, Jia-Bao Yan, Zhi-Wei Gong, Zhong-Hua Yang

**Affiliations:** 1 College of Chemistry and Chemical Engineering, Hubei University, Wuhan, 430062 China; 2 School of Chemical Engineering and Technology, Wuhan University of Science and Technology, Wuhan, 430081 China

**Keywords:** *Trichoderma virens*, Endo-1, 4-β-d-glucanase, Cellulose, Heterologous expression

## Abstract

Endo-1,4-β-d-glucanase (EG), as a key constituent of cellulase taking the responsibility of cutting β-1,4 glycosidic bonds, plays the essential role in the process of degrading cellulose by cellulase. Cloning and expressing the EG gene is important to the cellulase research and application. In this work, a novel EG gene was cloned from *Trichoderma virens* ZY-01, which was a cellulase secreting microbe isolated by our laboratory. The DNA sequence showed that the length of the cloned EG is 1069 bp, which had 95.2% similarity to the EG IV from *T. viride* AS 3.3711. Further, the expression vector pET-32a-EG was constructed and was successfully heterologously expressed in *Escherichia coli*. The expression product was purified with Ni^2+^ affinity chromatography and its enzymatic properties were investigated. The SDS-PAGE showed the target protein is 39 kDa, which is consistent with the translated result from the DNA sequence. The kinetic parameter for the expression product was K_m_ = 13.71 mg/mL and V_max=_0.51 μmol/min·mL. The optimal reaction pH and temperature was pH = 7.0 and T = 40 °C, which is similar to the native EG produced by *Trichoderma virens* ZY-01. It provides the foundation for the endo-1,4-β-d-glucanase further evolution and application.

**Electronic supplementary material:**

The online version of this article (doi:10.1186/s13568-016-0282-0) contains supplementary material, which is available to authorized users.

## Introduction

Cellulose, as a kind of renewable bioresource, is the most abundant biomass in nature. In the global world, the output of cellulose and hemicellulose is over 75 billion ton each year (Fang and Xia 2015a; Yücel and Aksu 2015). Hydrolysis of cellulose and hemicellulose to fermentable sugars is an economical and promising route for cellulose biomass utilization. Cellulase plays the key role in the route of cellulose utilization with biological technology (Fang and Xia 2015a). It is helpful to solve energy crisis, food shortage and environmental pollution.

Endo-1,4-β-d-glucanase (or endoglucanase, EG) is the major constituent of cellulase, which catalyzes the hydrolysis of the 1,4-β-d-glycosidic linkages in cellulose, hemicellulose, lichenin, and cereal β-d-glucans (Karlsson et al. 2001). The species of EG family is very numerous. In previous, some EG and its gene from various microbe were reported (Fang and Xia 2015b; Lhotak et al. 1988; Murray et al. 2003). Some EG gene was further overexpressed in *Saccharomyces cerevisiae* since *S. cerevisiae* preliminary post modification ability (Akcapinar et al. 2011; Huang et al. 2010; Qin et al. 2008; Wang and Zhang 2003). Also, some other constituent of cellulase have also been expressed in yeast system to improve its productivity (Barros and Thomson 1987; Fang and Xia 2013; Haan et al. 2007; Tang et al. 2009; Teng et al. 2007). However, discovery novel enzyme is always the eternal theme to enzyme research. In our previous work, we have isolated a novelty *Trichoderma virens* ZY-01, which can secrete high activity cellulase (Zeng et al. 2016). Especially, the EG enzyme activity is very outstanding. To clone the EG gene and express it in conventional heterologous host cell is very helpful to the cellulase research and its application.

In this work, an EG gene was cloned from the *T. virens* ZY-01 mRNA. Furthermore, it was expressed in *Escherichia coli* with pET-32a plasmid. Also, the enzymatic properties of the expression product (EG) were further investigated.

## Materials and methods

### Materials

Fungus *Trichoderma virens* ZY-01 (China patent ZL. 201210295819.6) was used for extract the total mRNA. This strain was isolated by our laboratory, which was collected in China Center for Type Culture Collection (CCTCC) with numbered M2012205 (Zeng et al. 2016). *E. coli* DH5α and *E. coli* BL21 (DE3) were respectively used for plasmid amplification and expression host cell. The pET-32a plasmid with ampicillin resistance was used as the cloning and expression vector. The modified Czapek medium was used for *T. virens* ZY-01 culture with composition as: NaNO_3_ 3 g, K_2_HPO_4_ 1 g, MgSO_4_ 0.5 g, KCl 0.5 g, FeSO_4_ 0.01 g, sucrose 20 g, CMC-Na 5 g, water 1 L. The Luria–Bertani medium was used for *E. coli* DH5α and *E. coli* BL21 (DE3) culture with composition as: tryptone 10 g, yeast extract 5 g, NaCl 10 g, water 1 L. The agar plate medium was the corresponding liquid medium with addition of 1.5% agar.

### Total RNA extraction from *T. virens* ZY-01

The total RNA of *T. virens* ZY-01 was extracted from its spores. The *T. virens* ZY-01 was inoculated on Czapek medium agar plate and incubated at 30 °C for 2–3 days. When the mycelium turn green and abundant spores appear, the green spores were collected and washed with ddH_2_O treated by DEPC. The collected spores were grinded in liquid nitrogen, and the mRNA of *T. virens* ZY-01 was extracted with RNAprep Pure Plant Kit (Tiangen Biotech, Beijing) according to the kit manual.

### EG gene clone from *T. virens* ZY-01

The EG gene was obtained by RT-PCR amplification using cDNA as the template,the corresponding signal peptide was removed. The cDNA was synthesized from the mRNA extracted from *T. virens* ZY-01 with the RevertAid First Strand cDNA Synthesis Kit (Thermo Scientific™). The primers for EG PCR were: Forward primer 5′-GCCATGGCTGATATCGGATCCCTGCTGGTTAC CGCTCTGGC-3′ (with *Bam*HI cleavage sites) and Reverse primer 5′-CTCGAGTGCGGCCGC AAGCTTGTTCAGGCACTGAGCGTAG-3′ (with *Hin*dIII cleavage sites). The PCR reaction conditions were: prepared denaturation at 95 °C for 5 min, 94 °C denaturation for 1 min, 59 °C annealing for 45 s, 72 °C extension for 90 s, repetition for 34 cycles, followed by final 72 °C extension for 10 min. The cloned EG gene was sequenced by Sangon Biotech (Shanghai) Co., Ltd and the EG gene nucleotide sequence was deposited in GenBank^®^ database with an accession number KX931112.

### Construction of *E. coli* BL21(DE3)/pET-32a-EG recombinant heterologous expression systems

The EG gene was inserted into plasmid pET-32a with ligation reaction between the digested EG PCR fragment and pET-32a by *Bam*HI and *Hin*dIII. After the insertion of EG gene into pET-32a, the recombinant plasmid pET-32a-EG was obtained, this was the expression vector. The expression vector pET-32a-EG was verified with colony PCR and double restriction endonuclease digestion. Furthermore, the EG gene was sequenced. To express EG gene, the vector pET-32a-EG was transformed into *E. coli* BL21(DE3) with electroporation, then the recombinant *E. coli* BL21(DE3)/pET-32a-EG was obtained.

### Expression and purification of recombinant EG in *E. coli* BL21(DE3)/pET-32a-EG

The *E. coli* BL21(DE3)/pET-32a-EG was inoculated in Luria–Bertani medium containing 100 μg/ml ampicillin and incubated at 37 °C. When OD_600nm_ reached to 0.4–0.6, IPTG (Isopropyl β-d-Thiogalactoside) was added to induce EG expression at 30 °C for 6 h. After culture, the cell was collected from the culture broth by centrifuge (10,000×*g* for 10 min). The cell sludge was washed with Tris–HCl buffer. To release the expression product, the cell was lysed sonication. After remove the cell debris with centrifuge, the crude EG enzyme solution was prepared. The EG was further purified by affinity chromatography with Ni^2+^ affinity resin (Profinity IMAC Resins, Bio-rad Shanghai, China).

### Evaluation of enzymatic properties

The following enzymatic properties of the EG expressed by the *E. coli* BL21(DE3)/pET-32a-EG were evaluated. The optimum pH, temperature and effects of metal ions to the enzyme activity were investigated. Also, the Michaelis–Menten kinetic constants (K_m_) and maximum velocity (V_max_) for substrate CMC-Na were determined based on the initial reaction rate and the model was fit with least squares. The general procedure for enzymatic properties investigation as following: 1 mL of enzyme solution was added into 1 mL reaction mixture containing 0.5% CMC-Na (Carboxymethylcellulose sodium) in citrate buffer (pH = 7.0), and incubated for 1 h at a given temperature 45 °C. After incubation, the reducing sugar (product) was measured and the EG enzyme activity was assayed. To evaluate the various effect factors on the enzyme activity, the corresponding factor was changed. The temperature range was from 30 to 90 °C, and pH was range from 2 to 12, the buffering systems at various pH were acetate buffer (pH 2.0–3.0), citrate buffer (pH 4.0–6.0), phosphate buffer (pH 6.0–8.0), Tris–Hcl buffer (pH 8.0–10.0), Glycine-NaoH buffer (pH 10.0), Kcl–NaoH buffer (pH 11.0–12.0), and the concentration of metal ions was 1 mmol/L. All of the above experiments were completed in triplicate, and average values were calculated based on results from the three independent experiments.

### EG activity assay

EG activity assay was based on the ability of catalyzing the hydrolysis of standard CMC-Na to reducing sugar (Dashtban et al. 2010). The assay for EG activity was carried out with mixing 1 mL of reaction mixture (containing 0.1% (w/v) CMC Na with pH 5.0 citric acid buffer) and 1.0 mL of enzyme solution and incubating at 50 °C for 1 h. Then the reducing sugar produced by the reaction was determined by 3,5-Dinitrosalicylic acid (DNS) method (Miller 1959). One unit of the EG enzyme activity was defined as the amount of enzyme that catalyzed to produce 1 μmol of reduced sugar per minute with the reduction of CMC-Na. The corresponding experiments were conducted in triplicate, and average values were calculated based on results from the three independent experiments.

## Results

### Cloning and sequence analysis of EG gene from *T. virens* ZY-01

The total RNA was extracted from the *T. virens* ZY-01 spores with RNAprep Pure Plant Kit after lysis the spores in liquid nitrogen. The RNA sample was detected with agarose gel electrophoresis, and the results indicated that the total RNA was pure and intact (the photography of *T. virens* ZY-01 spores and agarose gel of total RNA was respectively given in Additional file 1: Figures S1, S2).

**Fig. 1:**
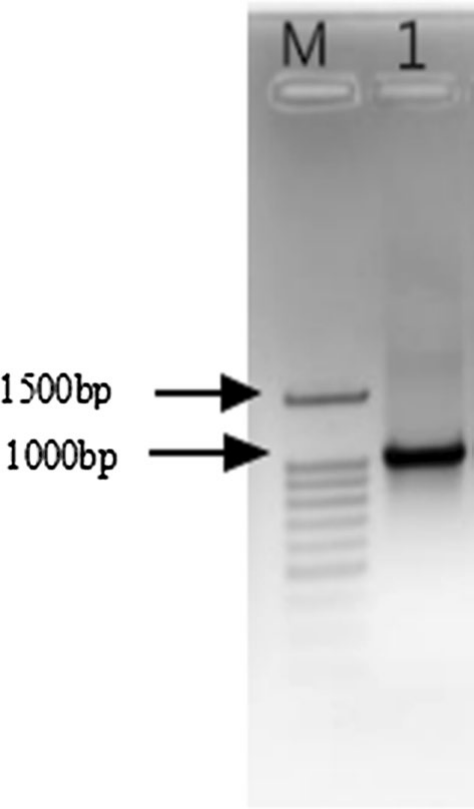
Agarose gel map of RT-PCR EG gene. *Lane M* DNA ladder, *Lane 1* RT-PCR product

The EG gene was cloned from *T. virens* ZY-01 total RNA by RT-PCR. The RT-PCR product was determined by agarose gel electrophoresis. The gel result, which given in Fig. 1, showed that the PCR product was single band and the product size was about 1010 bp. The DNA was sequenced by Sangon Biotech (Shanghai) Co., Ltd. The sequence result showed that the length of EG gene is 1069 bp, which encoded 356 aa. The nucleotide sequence was deposited in GenBank with an accession number KX931112. We conducted the BLAST on NCBI and found that this EG gene was very similar to the EG IV gene from *Trichoderma viride* strain AS 3.3711. The similarity for DNA was 95.2% (the detail information was given in Additional file 1: Figure S3). So it can be deduced that the cloned gene from *T. virens* ZY-01 is EG IV.

### Construction of *E. coli* BL21(DE3)/pET-32a-EG recombinant heterologous expression systems

**Fig. 2:**
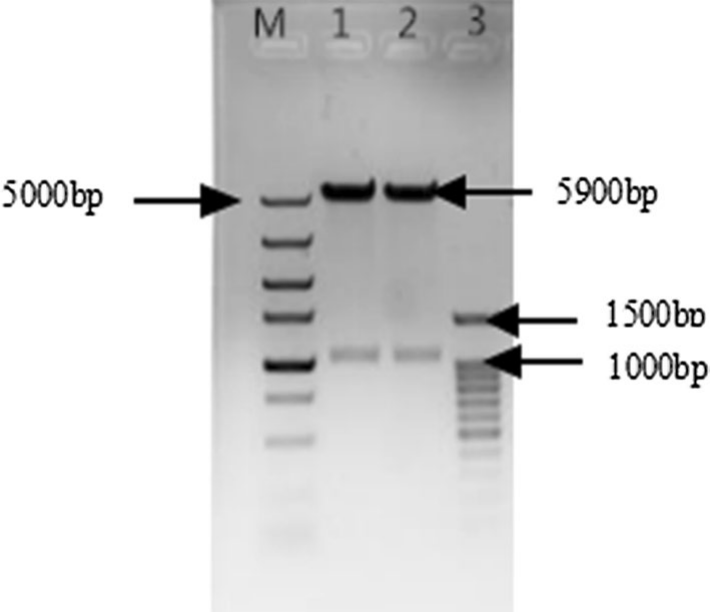
Agarose gel map of double enzyme digestion of extracted plasmid. *Lane M* DNA ladder, *Lane 1* and *2* double enzyme digestion product, *Lane 3* DNA ladder

To express the EG in *E. coli*, an efficient expression vector containing the EG gene is essential. The corresponding vector was constructed through insertion of the EG gene fragment into pET-32a with ligase using the digested EG gene fragment and plasmid by *Bam*HI and *Hin*dIII. To verify the recombinant plasmid pET-32a-EG, it was used to transform *E. coli* DH5α for plasmid amplification. The clony PCR for the transformant was used to preliminarily verify the plasmid pET-32a-EG. The agarose gel for the clony PCR was shown in Additional file 1: Figure S4. It showed that the EG gene was insert into *E. coli* DH5α. In order to further confirm the pET-32a-EG vector, the plasmid was extracted from the transformant and double digested with *Bam*HI and *Hin*dIII. Figure 2 was the agarose gel for the digestion product. The gel showed that there were two bands, one was about 5900 bp, and the other was about 1060 bp, they were respectively for the plasmid pET-32a and EG gene. This evidenced that the expression vector pET-32a-EG was successfully constructed.

To express the EG, the vector pET-32a-EG was transformed into *E. coli* BL21(DE3) by electroporation. The transformant, *E. coli* BL21(DE3)/pET-32a-EG, was selected with LB medium agar plate with ampicillin.

### Expression of EG IV gene in *E. coli* BL21(DE3)/pET-32a-EG

**Fig. 3:**
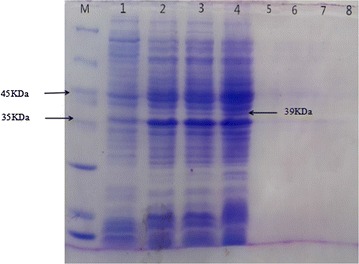
SDS-PAGE of the EG expressed *E. coli* BL21(DE3)/pET-32a-EG. *Lane M* protein ladder, *lane 1* control with blank plasmid cell sediment induced by 1.0 mmol/L IPTG, *lane 2* cell sediment induced by 0.4 mmol/L IPTG, *lane 3* cell sediment induced by 0.7 mmol/L IPTG, *lane 4* cell sediment induced by 1.0 mmol/L IPTG, *lane 5* control with blank plasmid supernatant induced by 1.0 mmol/L IPTG, *lane 6* cell sediment induced by 0.4 mmol/L IPTG, *lane 7* supernatant induced by 0.7 mmol/L IPTG, *lane 8* supernatant induced by 1.0 mmol/L IPTG

**Fig. 4:**
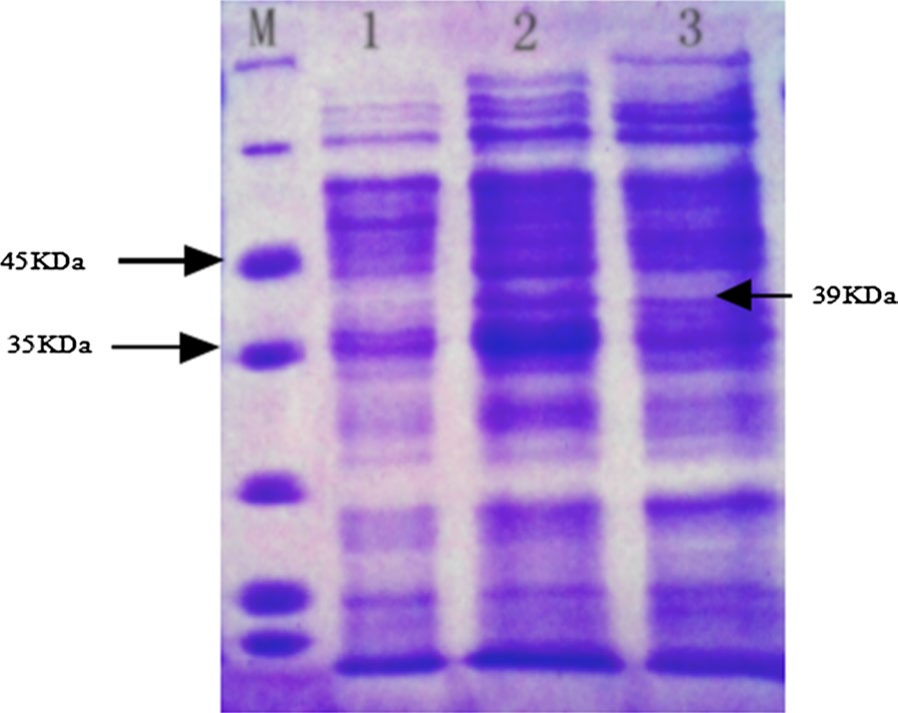
SDS-PAGE of supernatant and cell debris after cell lysing. *Lane M* protein ladder, *lane 1* control with blank plasmid cell supernatant, *lane 2* the supernatant after cell lysing, *lane 3* the cell debris after cell lysing

The EG was expressed by *E. coli* BL21(DE3)/pET-32a-EG in Luria–Bertani medium with IPTG. The supernatant and cell was collected from the culture broth by centrifugation. The supernatant and sediment were respectively disposed with SDS-PAGE loading buffer and boiled at 100 °C for 5 min. Then they were detected with SDS-PAGE. The results were given in Fig. 3. Based the EG gene DNA sequence, the aa of EG can be deduced. The expression product would be about 39 kDa. The SDS-PAGE showed that the EG was expressed in *E. coli* BL21(DE3)/pET-32a-EG was also about 39 kDa. The supernatant and cell debris of recombinant cells after lysing were detected by SDS-PAGE, Fig. 4 is the gel results. Comprehensive analyzing of the results Figs. 3 and 4, it verified that the target protein is an intracellular enzyme. It was not secreted to the extracellular broth. Overcoming the solubility problem of expression eukaryotes protein in *E. coli* is always a challenge (Correa and Oppezzo 2015). In the paper, we adopted a soluble vector pET-32a, and the target protein was detected in supernatant after lysing cells. This verified that pET-32a is a suitable vector for *T. virens* EG expression.

**Fig. 5:**
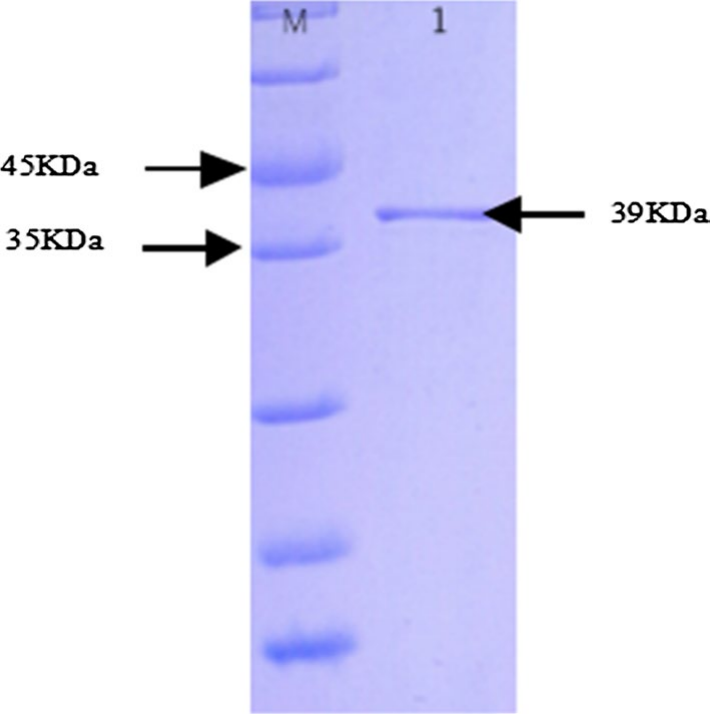
SDS-PAGE for purified EG expression with affinity chromatography. *Lane M* protein ladder, *lane 1* purified expression product EG

In order to purify the recombinant EG, the affinity chromatography with Ni^2+^ affinity resin was used since the expression product with pET-32a contains a His-tag. The EG was collected from the eluent. Figure 5 is the SDS-PAGE for the purified product. The targeted band was a single band with 39 kDa, which is consistent with the result deduced from the DNA sequence. Li et al. reported cloning EG I gene from *T. viride* and expressed in *Bombyx Mori*, the target protein was about 49 kDa (Li et al. 2010). Huang et al. reported an EG VIII from *T. viride* AS3.3711, encoding a 438 amino acid protein with 46.86 kDa of molecular mass (Huang et al. 2010). This shows that the similar constituent of EG was with different molecular mass.

### Properties of EG expressed by *E. coli* BL21(DE3)/pET-32a-EG

**Fig. 6:**
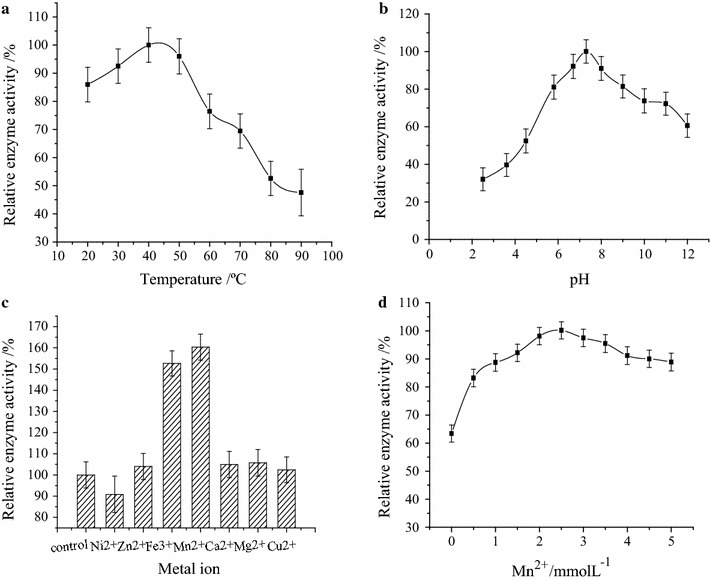
Various factors effect on the EG activity. **a** Reaction temperature, **b** reaction pH, **c** metal ion, **d** Mn^2+^ concentration

The enzymatic properties are the fundamental enzymology data to an enzyme research and application. They are essential to its application. Firstly, the enzymatic properties such as effect of reaction temperature, pH and metal ions to enzyme activity were investigated. Furthermore, the kinetic parameters of the EG expressed by *E. coli* BL21(DE3)/pET-32a-EG was also determined. The results of reaction temperature, pH, metal ions to enzyme activity and the enzymatic stability were presented in Fig. 6. The relative enzyme activity was applied to indicate the effect, which was defined as the highest enzyme as 100% relative enzyme activity.

The results showed that the EG activity was highest at 40 °C, which was lower than the EG gene expressed in silkworm cell line (Li et al. 2010). When further increased the reaction temperature, the enzyme activity would sharply decrease due to the EG thermal inactivation. To pH condition, the EG can obtain the highest enzyme activity at pH 7.0, so the optimum reaction pH is 7.0, which was same to the EG expressed in silkworm cell line (Li et al. 2010). The metal ions and its concentration may influence the enzyme activity, the research result showed that the Mn^2+^ and Fe^3+^ could significantly activate the EG activity, while Ni^2+^ slightly inhibit the enzyme activity. The appropriate Mn^2+^ concentration was 2.5 mmol/L, with this concentration the highest EG activity can reached.

The kinetic parameters of the EG expressed by *E. coli* BL21(DE3)/pET-32a-EG was evaluate based on Michaelis–Menten model. The K_m_ and V_max_ to substrate CMC-Na was calculated by fitting the model with least squares. V_max_ is 0.51 μmol/min·mL and K_m_ is 13.71 mg/mL. The fitting curve was showed in Additional file 1: Figure S5.

## Discussion

In order to find another more effective gene encoding endoglucanase, a novel endo-β-1, 4-glucanase gene from *T. virens* was cloned and successfully expressed in *E. coli* BL21 (DE3) in this paper. Nowadays, it is very important to produce bioethanol as a fuel from recycling of biomass resources. To exploit large quantities of cellulase with high bioactive efficiency is important to the bioethanol industry, which can efficiently hydrolyze cellulose biomass to fermentable sugar (Zaldivar et al. 2001). The cellulase was mainly secreted from eukaryotic microbe. Heterologous expression is an efficient route to improve cellulase productivity. There may be many problems to express gene from eukaryotic organism in procaryotic organism (Barros and Thomson 1987) especially applying the most common *E. coli* as the host cell (Akcapinar et al. 2011). However, prokaryotic expression system is a mature system; also it is easy to be cultured and high productivity (Tang et al. 2009). However, we adopted the plasmid pET-32a, which contained an extra label encoding thioredoxin to help disulfide bond folded correctly.

The enzymatic properties are the fundamental bioinformation for enzyme production and application. Temperature could speed up the reaction, but the activity of recombinant endoglucanase would fade along with the increasing temperature (Andreaus et al. 1999). The results showed that the optimal temperature for EG in this work is about 40 °C, it was lower than the endoglucanase expressed in silkworm cell line (Li et al. 2010). But the optimal temperature is obvious higher than the crude enzyme production by *Trichoderma reesei* on straw substrate, which was about 27 °C (Rosyida et al. 2015). The pH value can affect the enzyme structure and its activity, the optimal pH value for the EG expressed in this work is same to which expressed in silkworm cell line (Li et al. 2010).

In summary, we have successfully cloned the EG gene from *T. virens* ZY-01 through RT-PCR, and constructed the expression vector plasmid pET32a-EG. The EG was effectively expressed in *E. coli* BL21(DE3)/pET32a-EG. The SDS-PAGE result showed that the target protein was soluble intracellular enzyme. The EG enzyme activity in various condition was determined. In present paper, the recombinant EG exhibited a high specificity and hydrolysis capacity against CMC. These advantages make it a very potential application in industry.

## Additional file

**Additional file 1: Figure S1.** Green spores of *T. viride* ZY-01. **Figure S2**. Agarose gel of extracted RNA from *T. viride* ZY-01. **Figure S3**. EG gene Nucleotide sequence blast between strain *T. virens* ZY-01 and *T. viride* AS3.3711. **Figure S4**. Agarose gel map of *E. coli* DH5α/pET-32a-EG clony PCR. **Figure S5**. The fitting curve of Michaelis–Menten model.

## References

[CR1] Akcapinar GB, Gul O, Sezerman U (2011). Effect of codon optimization on the expression of *Trichoderma reesei* endoglucanase I in *Pichia pastoris*. Biotechnol Prog.

[CR2] Andreaus J, Azevedo H, Paulo AC (1999). Effects of temperature on the cellulose binding ability of cellulase enzymes. J Mol Catal B Enzym.

[CR3] Barros ME, Thomson JA (1987). Cloning and expression in *Escherichia coli* of a cellulase gene from *Ruminococcus flavefaciens*. J Bacteriol.

[CR4] Correa A, Oppezzo P, Elena GF (2015). Overcoming the solubility problem in *E. coli*: available approaches for recombinant protein production. Insoluble Proteins, volume 1258 of the series methods in molecular biology.

[CR5] Dashtban M, Maki M, Leung KT, Mao C, Qin W (2010). Cellulase activities in biomass conversion measurement methods and comparison. Crit Rev Biotechnol.

[CR6] Fang H, Xia L (2013). High activity cellulase production by recombinant *Trichoderma reesei* ZU-02 with the enhanced cellobiohydrolase production. Bioresour Technol.

[CR7] Fang H, Xia L (2015). Cellulase production by recombinant *Trichoderma reesei* and its application in enzymatic hydrolysis of agricultural residues. Fuel.

[CR8] Fang H, Xia L (2015). Heterologous expression and production of *Trichoderma reesei* cellobiohydrolase II in *Pichia pastoris* and the application in the enzymatic hydrolysis of corn stover and rice straw. Biomass Bioenergy.

[CR9] Haan RD, Mcbride JE, Grange DCL, Lynd LR, Zyl WHV (2007). Functional expression of cellobiohydrolases in *Saccharomyces cerevisiae* towards one-step conversion of cellulose to ethanol. Enzyme Microb Technol.

[CR10] Huang XM, Yang Q, Liu ZH, Fan JX, Chen XL, Song JZ, Wang Y (2010). Cloning and heterologous expression of a novel endoglucanase gene egVIII from *Trichoderma viride* in *Saccharomyces cerevisiae*. Appl Biochem Biotechnol.

[CR11] Karlsson J, Saloheimo M, Siika-aho M (2001). Homologous expression and characterization of Cel61A(EG IV) of *Trichoderma reesei*. Eur J Biochem.

[CR12] Lhotak P, Moravek J, Smejkal T, Stibor I, Sykora J (1988). EGIII, a new endoglucanase from *Trichoderma reesei*: the characterization of both gene and enzyme. Gene.

[CR13] Li XH, Wang D, Zhou F, Yang HJ, Bhaskar R, Hu JB, Sun CG, Miao YG (2010). Cloning and expression of a cellulase gene in the silkworm, *Bombyx mori* by improved Bac-to-Bac/BmNPV baculovirus expression system. Mol Biol Rep.

[CR14] Miller GL (1959). Use of dinitrosalicylic acid reagent for determination of reducing sugar. Anal Chem.

[CR15] Murray PG, Collins CM, Grassick A, Tuohy MG (2003). Molecular cloning, transcriptional, and expression analysis of the first cellulase gene (cbh2), encoding cellobiohydrolase II, from the moderately thermophilic fungus *Talaromyces emersonii* and structure prediction of the gene product. Biochem Biophys Res Commun.

[CR16] Qin Y, Wei X, Liu X, Wang T, Qu Y (2008). Purification and characterization of recombinant endoglucanase of *Trichoderma reesei* expressed in *Saccharomyces cerevisiae* with higher glycosylation and stability. Protein Expr Purif.

[CR17] Rosyida VT, Indrianingsih AW, Maryana R, Wahono SK (2015). Effect of temperature and fermentation time of crude cellulase production by *Trichoderma reesei* on straw substrate. Energy Procedia.

[CR18] Tang B, Pan H, Zhang Q, Ding L (2009). Cloning and expression of cellulase gene EGI from *Rhizopus stolonifer* var. reflexus TP-02 in *Escherichia coli*. Bioresour Technol.

[CR19] Teng D, Fan Y, Yang YL, Tian ZG, Luo J, Wang JH (2007). Codon optimization of bacillus licheniformis beta-1,3-1,4-glucanase gene and its expression in *pichia pastoris*. Appl Microbiol Biotechnol.

[CR20] Wang LS, Liu J, Zhang YZ (2003). Comparison of domains function between cellobiohydrolase I and endoglucanase I from *Trichoderma* pseudokoningii S-38 by limited proteolysis. J Mol Catal B Enzym.

[CR21] Yücel HG, Aksu Z (2015). Ethanol fermentation characteristics of *Pichia stipitis* from sugar beet pulp hydrolysate: use of new detoxification methods. Fuel.

[CR22] Zaldivar J, Nielsen J, Olsson L (2001). Fuel ethanol production from lignocellulose: a challenge for metabolic engineering and process integration. Appl Microbiol Biotechnol.

[CR23] Zeng R, Yin XY, Ruan T, Hu Q, Hou YL, Zuo ZY, Huang H, Yang ZH (2016). A novel cellulase produced by a newly isolated *Trichoderma virens*. Bioengineering.

