# Supplementary material for: Cloning a novel endo-1,4-β-d-glucanase gene from Trichoderma virens and heterologous expression in E. coli
Source: AMB Express. 2016 Nov 9;6:108. doi: 10.1186/s13568-016-0282-0 (PMC5103005; doi:10.1186/s13568-016-0282-0)

Supplementary Materials

AMB Express

Tittle: Cloning a novel endo-1,4-β-D-glucanase gene from *Trichoderma virens* and heterologous expression in *E. coli*

Rong Zeng[1], Qiao Hu[2], Xiao-YanYin[2], Hao Huang[2], Zhong-Hua Yang[2]*

[1]College of Chemistry and Chemical Engineering, Hubei University, Wuhan 430062, China;

[2]School of Chemical Engineering and Technology, Wuhan University of Science and Technology, Wuhan 430081, China

Corresponding author: Zhong-Hua Yang, E-mail: yangzh@wust.edu.cn Tel: +86-27-86563448

Figure S1

Green spores of *T. viride* ZY-01

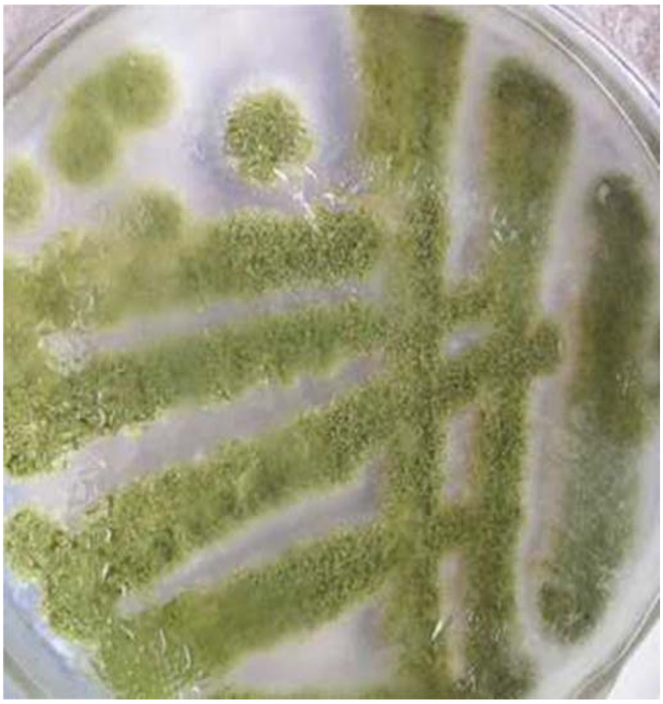

Figure S2

Agarose gel of extracted RNA from *T. viride* ZY-01.

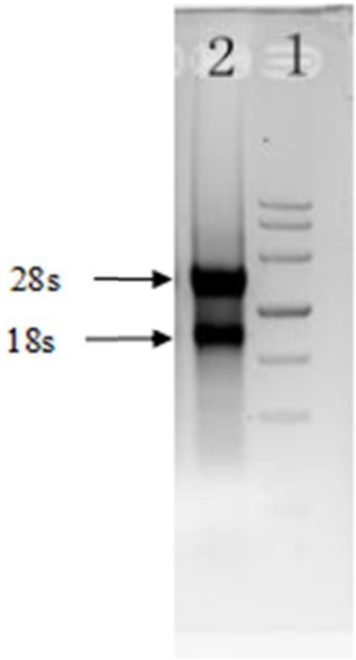

Lane 1 DNA ladder, Lane 2 total RNA sample

Figure S3

EG gene Nucleotide sequence blast between strain *T. virens* ZY-01and *T. viride* AS3.3711

ZY-01 1 --CTCCTTGTCACCGCACTGGCTGTTGCTACCGGCGTTGTT 60

AS 3.3711 1 ATGATCCAGAAGCTTTCCAACCTCCTTGTCACCGCACTGGCGGTGGCTACTGGCGTTGTC 60

ZY-01 61 GGACATGGTCATATTAATGACATTGTTATCAACGGGGTGTGGTACCAGGCCTATGACCCT 120

AS 3.3711 61 GGACATGGACATATTAATGACATTGTCATCAACGGGGTGTGGTATCAGGCCTATGATCCT 120

ZY-01 121 ACAACCTTTCCATACGAATCAAACCCCCCCATCGTAGTGGGCTGGACCGCTGCCGACCTG 180

AS 3.3711 121 ACAACGTTTCCATACGAGTCAAACCCCCCCATAGTAGTGGGCTGGACGGCTGCCGACCTT 180

ZY-01 181 GACAACGGTACGTGATCCTCATCTCTATCTGTACAACGCTCATGCTAATCCAACTCAATA 240

AS 3.3711 181 GACAACGGTACGTGATCCTCATCTCTATCTGTACAACGCTCATGCTAATCCAACTCAATA 240

ZY-01 241 GGCTTCGTTTCACCGGACGCATACCAGAACCCTGACATCATCTGCCACAAAAATGCTACG 300

AS 3.3711 241 GGCTTCGTTTCACCCGACGCATACCAAAACCCTGACATCATCTGCCACAAGAATGCTACG 300

ZY-01 301 AATGCCAAAGGGCACGCTTCTGTTAAGGCCAGTGACACTATTCTGTTCCAGTGGGTTCCA 360

AS 3.3711 301 AATGCCAAGGGGCACGCGTCTGTCAAGGCCAGAGACACTATTCTCTTCCAGTGGGTGCCA 360

ZY-01 361 GTTCCGTGGCCGCACCCTGGTCCCATTGTCGACTACCTGGCCAACTGCAATGGTGACTGC 420

AS 3.3711 361 GTTCCATGGCCGCACCCTGGTCCCATTGTCGACTACCTGGCCAACTGCAATGGTGACTGC 420

ZY-01 421 GAAACCGTTGACAAGACCACGCTGGAGTTCTTCAAAATCGATGGCGTTGGTCTGCTCAGC 480

AS 3.3711 421 GAGACCGTTGACAAGACGACGCTTGAGTTCTTCAAGATCGATGGCGTTGGTCTCCTCAGC 480

ZY-01 481 GGCGGGGACCCGGGCACCTGGGCCTCTGACGTTCTGATCTCCAACAACAACACCTGGGTT 540

AS 3.3711 481 GGCGGGGATCCGGGCACCTGGGCCTCAGACGTGCTGATCTCCAACAACAACACCTGGGTC 540

ZY-01 541 GTCAAAATCCCCGACAACCTTGCGCCAGGCAATTACGTGCTCCGCCACGAAATCATCGCG 600

AS 3.3711 541 GTCAAGATCCCCGACAATCTTGCGCCAGGCAATTACGTGCTCCGCCACGAGATCATCGCG 600

ZY-01 601 TTGCACAGCGCCGGTCAGGCAAACGGTGCTCAGAACTACCCGCAGTGCTTCAACATTGCC 660

AS 3.3711 601 TTACACAGCGCCGGGCAGGCAAACGGCGCTCAGAACTACCCCCAGTGCTTCAACATTGCC 660

ZY-01 661 GTCTCTGGCTCTGGTTCTCTGCAGCCCAGCGGTGTTCTAGGGACCGACCTCTATCACGCG 720

AS 3.3711 661 GTCTCAGGCTCGGGTTCTCTGCAGCCCAGCGGCGTTCTAGGGACCGACCTCTATCACGCG 720

ZY-01 721 ACCGACCCTGGTGTTCCGATCAACATCTACACCAGCCCGCTGAACTACATCATCCCTGGA 780

AS 3.3711 721 ACGGACCCTGGTGTTCCCATCAACATCTACACCAGCCCGCTCAACTACATCATCCCTGGA 780

ZY-01 781 CCTACCGTTGTATCAGGCCTGGCCAACGAGTGTTGCCCAGGGGAGCTCCGCCGCGACGGC 840

AS 3.3711 781 CCTACCGTGGTATCAGGCCTGGCCAACGAGTGTTGCCCAGGGGAGCTCCGCCGCGACGGC 840

ZY-01 841 CACCGCCAGCGCCACTGCTCCTGGAGGCGGTAGCGGCCCGACCAGCAGAACCACGACAAC 900

AS 3.3711 841 CACCGCCAGCGCCACTGCTCCTGGAGGCGGTAGCGGCCCGACCAGCAGAACCACGACAAC 900

ZY-01 901 GGCGAGGACGACCCAGGCCTCAAGCAGGCCCAGCTCTACGCCGCCCGCAACCACGTCGGC 960

AS 3.3711 901 GGCGAGGACGACGCAGGCCTCAAGCAGGCCCAGCTCTACGCCTCCCGCAACCACGTCGGC 960

ZY-01	961	ACCTGCTGGTGGCCCAACCCAGACTCTGTACGGCCAGTGTGGTGGTAGCGGTTACAGCGG	1020
AS 3.3711	961	ACCTGCTGGCGGCCCAACCCAGACTCTGTACGGCCAGTGTGGTGGCAGCGGTTACAGCGG	1020
ZY-01	1021	TCCTACCCGATGCGCTCCGCCAGCCACTTGCTCTACCTTGAACCCCTACTACGCCCAGTG	1080
AS 3.3711	1021	GCCTACCCGATGCGCGCCGCCAGCCACTTGCTCTACCTTGAACCCCTACTACGCCCAGTG	1080
ZY-01	1081	CCTTAACTAG	1090
AS 3.3711	1081	CCTTAACTAG	1090

Figure S4

Agarose gel map of *E. coli* DH5α/pET-32a-EG clony PCR

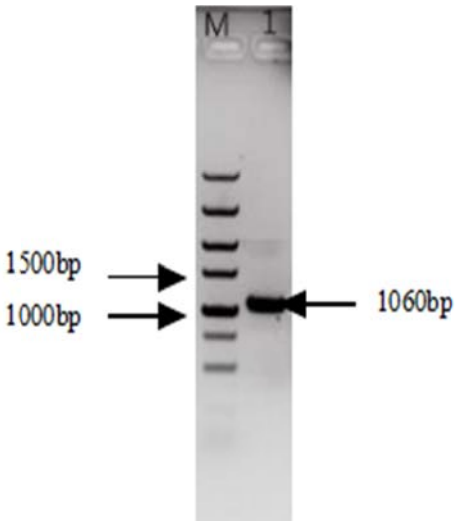

Lane M: DNA ladder, Lane 1: clony PCR product

Figure S5

The fitting curve of Michaelis–Menten model

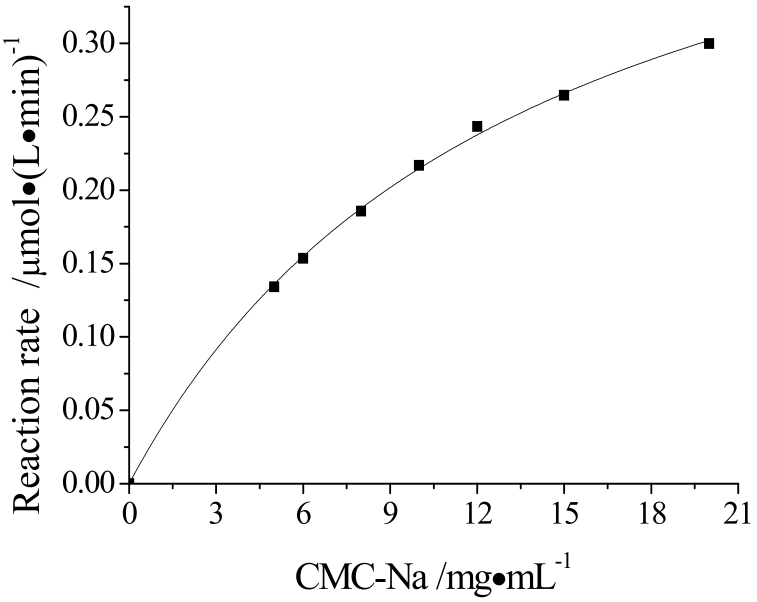

Supplement: Supplementary file 1 — Additional file 1: Figure S1. Green spores of T. viride ZY-01. Figure S2. Agarose gel of extracted RNA from T. viride ZY-01. Figure S3. EG gene Nucleotide sequence blast between strain T. virens ZY-01 and T. viride AS3.3711. Figure S4. Agarose gel map of E. coli DH5α/pET-32a-EG clony PCR. Figure S5. The fitting curve of Michaelis–Menten model. [file 13568_2016_282_MOESM1_ESM.pdf]
